# A Wearable Triboelectric Nanogenerator Based on Polyester‐Paper Cloth for Mechanical Energy Harvesting and Running Motion Sensing

**DOI:** 10.1002/open.202400373

**Published:** 2024-11-29

**Authors:** Xu Deng

**Affiliations:** ^1^ Henan University of Science and Technology Luoyang, Henan 471000 China

**Keywords:** Triboelectric nanogenerators (TENGs), Polyester-paper cloth, Self-powered sensor, Running motion, Intelligence sports

## Abstract

Paper‐based triboelectric nanogenerators (P‐TENGs) have recently garnered significant attention in wearable electronics. However, traditional P‐TENGs are constrained by the inherent strength limitations of paper. Hence, we reported a novel polyester‐paper cloth‐based triboelectric nanogenerator (PP‐TENG) designed for mechanical energy harvesting and running motion monitoring. Compared to paper, polyester‐paper cloth has higher durability and tear resistance. The PP‐TENG capitalizes on the unique fluffy internal structure of polyester‐paper cloth, imparting high sensitivity to pressure variations. Experimental results demonstrate that the PP‐TENG achieves an open‐circuit voltage (*V_oc_*) of 466.64 V, a short‐circuit current (*I_sc_*) of 48.73 μA, and a transfer charge (*Q_sc_*) of 90 nC. Its maximum output power reaches 930.26 μW when connected to a 40 MΩ load. These impressive metrics underscore the potential of PP‐TENG in energy harvesting applications, particularly for wearable electronic devices. The device's integration into the soles of athletic socks showcases its practical utility, providing real‐time monitoring of runners’ gait and step count. This integration not only enhances the functionality of sportswear but also offers valuable data for performance analysis and injury prevention, marking a significant advancement in wearable technology and intelligent textiles. This research provide a promising path for self‐powered wearable sensors and flexible electronics applications.

## Graphical abstract

This study introduces a polyester‐paper cloth‐based triboelectric nanogenerator (PP‐TENG) for efficient energy harvesting and real‐time running motion sensing. The PP‐TENG, integrated into athletic socks, leverages its durability and sensitivity to monitor gait and step count, demonstrating potential for wearable electronics and smart sportswear applications with high output and stability.

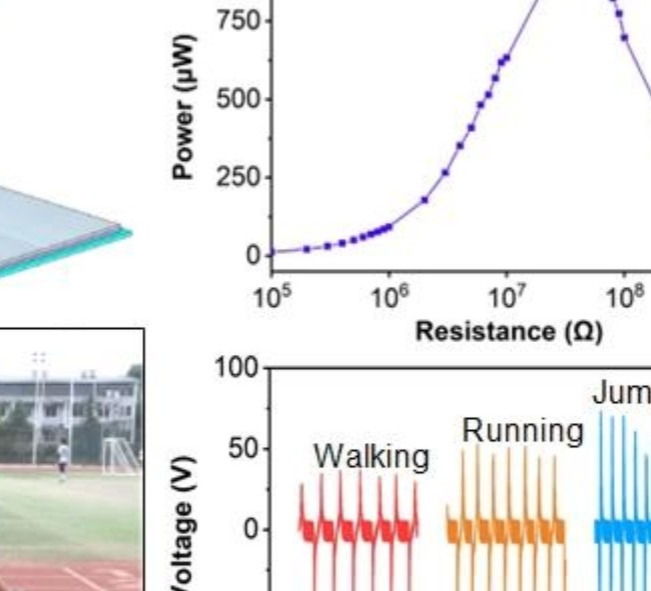

## 1 Introduction

As global energy consumption continues to rise, traditional centralized power generation methods–such as thermal, solar, wind, biomass, and hydropower–are becoming increasingly insufficient to meet the evolving demands of modern society.[^1^, ^2^] In response to carbon reduction goals, researchers worldwide are actively exploring innovative green alternative energy sources.^[3]^ Concurrently, the rise of intelligent sports and running exercises presents new opportunities for energy harvesting and distributed sensing.[^4^, ^5^] Self‐powered wearable electronic devices and sensors, which enable real‐time health monitoring while simultaneously harvesting energy from the environment, are increasingly being applied in these activities.[^6^, ^7^] An ideal bio‐mechanical energy harvesting device should be cost‐effective, lightweight, biocompatible, and environmentally friendly. However, traditional electromagnetic generators (EMGs), due to their substantial weight and volume, struggle to reach the rectifier's threshold voltage when their size is reduced, limiting their energy harvesting efficiency.^[8]^ Similarly, piezoelectric nanogenerators (PENGs) and thermoelectric generators (TEGs) face limitations when applied in wearable electronics.[^9^, ^10^] While PENGs utilize the piezoelectric effect to convert mechanical energy into electrical energy, they are constrained by limited material choices, low output power density, and issues related to fatigue and aging over extended use. TEGs, which generate electricity through temperature differences, are limited by the magnitude and stability of the temperature gradient, making it challenging to provide continuous and stable power in practical applications. Triboelectric nanogenerators (TENGs), first proposed by Wang and colleagues, have revolutionized the field of bio‐mechanical energy harvesting.[^11^, ^12^, ^13^, ^14^, ^15^, ^16^, ^17^, ^18^, ^19^, ^20^, ^21^] Operating on the principles of contact electrification and electrostatic induction, TENGs efficiently capture low‐frequency mechanical energy, making them capable of harvesting a wide range of ubiquitous and abundant mechanical energy sources.[^22^, ^23^] TENGs have garnered significant attention due to their simple fabrication process, cost‐effectiveness, lightweight design, diverse material compatibility, high power output density, and efficient energy conversion capabilities. Compared to piezoelectric nanogenerators (PENGs) and thermoelectric generators (TEGs), TENGs offer distinct advantages in terms of lightweight materials and multi‐functionality.^[24]^ They are highly adaptable to various complex environments, maintaining efficient energy conversion even under low‐frequency mechanical motion. Significant progress has been made in TENG research, including advancements in structural designs tailored for specific applications,^[25]^ triboelectric material combinations,^[26]^ output optimization,^[27]^ power management circuits,^[28]^ and specialized applications.^[29]^ Despite these achievements, selecting optimal triboelectric materials remains a critical challenge, limiting large‐scale industrial production and commercial adoption of TENGs. Nevertheless, TENGs hold great potential for wearable electronic devices, and with continuous technological advancements and optimization, they are poised for broader applications and significant breakthroughs in the near future.

Human motion often involves considerable deformation, making bio‐mechanical energy harvesters with high flexibility and toughness more suitable for practical applications. Flexible triboelectric nanogenerators (TENGs) can effectively harness both environmental and mechanical energy through various mechanisms, which has attracted sustained attention from related industries.^[30]^ Previous research efforts have focused on developing flexible TENGs to harvest bio‐mechanical energy.[^31^, ^32^] However, to meet the dual demands of flexibility and environmental sustainability in real‐world applications, further improvements in both flexibility and eco‐friendliness are necessary. A versatile energy harvester that can be worn on the skin or embedded into clothing holds great promise in this regard. Among the various flexible energy harvesters, paper‐based TENGs have emerged as highly promising due to their unique material properties and structural design.[^33^, ^34^, ^35^, ^36^] Paper offers several advantages, including light weight, low cost, high flexibility, and biocompatibility, making it an ideal material for wearable devices. Additionally, paper‐based TENGs exhibit stable output under different deformation conditions and can achieve multifunctional integration through simple processing techniques. Moreover, their electrical performance can be further enhanced through surface treatments and coating methods.[^37^, ^38^] Various materials have been explored as flexible components for TENGs, such as thermoplastic polyurethane (TPU) films,^[39]^ chitosan films,^[40]^ liquid metal,^[41]^ and ionic hydrogels.^[42]^ In comparison, paper‐based TENGs circumvent issues like liquid leakage, offering better stability and durability. Furthermore, their manufacturing process is straightforward, with abundant material sources, aligning with the goals of green and flexible electronics development. Therefore, paper‐based TENGs hold significant potential for application and research in flexible electronics and wearable energy harvesting devices.

In this study, we propose a polyester‐paper cloth triboelectric nanogenerator (PP‐TENG) designed for mechanical energy harvesting and running monitoring. The triboelectric pair consists of polyester‐paper cloth and PTFE tape, integrated onto a flexible substrate. Due to the weak electron affinity of the polyester‐paper cloth, it functions as a positive triboelectric material. The performance of the PP‐TENG is demonstrated by an open‐circuit voltage (V_oc_) of 466.64 V, a short‐circuit current (I_sc_) of 48.73 μA, and a transfer charge (Q_sc_) of 90 nC. Furthermore, the device achieves a maximum output power of 930.26 μW with an optimal load of 40 MΩ, indicating its high efficiency and strong potential for energy harvesting applications. The unique fluffy internal structure of the polyester‐paper cloth contributes to the PP‐TENG's heightened sensitivity to pressure, enabling its seamless integration into the soles of athletic socks. This integration provides a robust platform for gait and step count monitoring, offering valuable data for running training analysis and injury prevention in athletes. Thus, the PP‐TENG represents a promising innovation in wearable technology and smart textiles, enhancing both functionality and performance in energy harvesting and monitoring applications.

## Experimental Section

### Materials

The flexible plastic sheets were bought from Shanghai Rourou New Technology Co., Ltd. The conductive aluminum foil was purchased from Shanghai Lingguan New Materials Co., Ltd. PTFE tape was obtained from Taixing Chuangwei Composite Materials Co., Ltd. The polyester‐paper cloth was purchased from Jinan Shengquan Group Co., Ltd. Flexible substrates, conductive electrodes, triboelectric layers, etc. are pasted with double‐sided adhesive and compacted by mechanical presses.

### Fabrication of PP‐TENG

**Figure 1:**
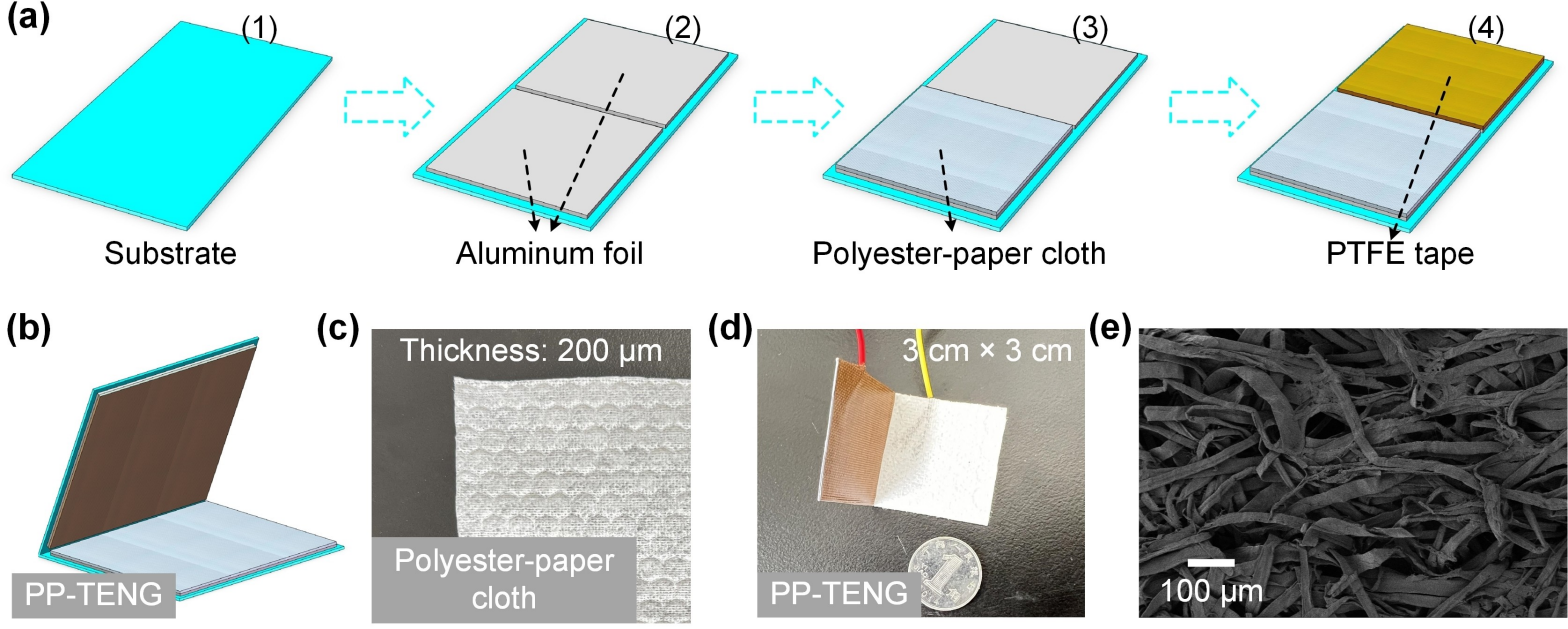
(a1–a4) The simple preparation method for PP‐TENG device. (b) Schematic diagram of PP‐TENG device. The picture of (c) polyester‐paper cloth and (d) PP‐TENG device. (e) The SEM image of polyester‐paper cloth surface.

In this design, the preparation method of PP‐TENG was very simple and suitable for large‐scale production, as exhibited in Figure 1(a1–a4). Specifically, flexible plastic sheets were cut into rectangular substrates to serve as the load‐bearing material for the entire PP‐TENG device (Figure 1(a1)). The flexible substrate adapted to various deformation environments and restored its original state after deformation. Furthermore, conductive aluminum foil was pasted on the surface of the flexible substrate as the conductive electrode of PP‐TENG to provide electrical energy output for external loads (Figure 1(a2)). Then, a layer of polyester‐paper cloth was stuck on the surface of the other electrode as a positive triboelectric material (Figure 1(a3)). In addition, a layer of PTFE tape was pasted on the surface of the aluminum electrode as a negative triboelectric material (Figure 1(a4)). Finally, the flexible base was folded in half to form a Z‐shaped structure, which achieved good response to mechanical motion due to its excellent elasticity (Figure 1(a5)).

### Characterizations and Measurements

Figure 1(b) presented a picture of the polyester‐paper cloth, and it could be observed that there were many patterns on the surface of the polyester‐paper cloth. These fluffy textures contributed to the generation and accumulation of triboelectric charges. The physical image of PP‐TENG was illustrated in Figure 1(c), and a coin was used as a size reference to evaluate the size of PP‐TENG. In Figure 1(d), the image shows a physical prototype of the PP‐TENG device. The PP‐TENG is shown next to a coin, which provides a size reference for the device, indicating its compact dimensions. The PP‐TENG is composed of layered materials, including polyester‐paper cloth and PTFE tape, as described in the fabrication process. The wires attached to the device suggest it is ready for testing or energy harvesting applications. In Figure 1(e), a scanning electron microscopy (SEM) image of the polyester‐paper cloth is displayed. The micrograph reveals the rough and fibrous structure of the material. This microstructure, with its intricate network of fibers, is advantageous for enhancing contact electrification, thereby contributing to the triboelectric charge generation capabilities of the PP‐TENG. The scale bar indicates a size of 100 μm, providing a sense of the fine detail in the material's surface. Moreover, the Keithley 6514 electrometer was used to test the *I_sc_* and *Q_sc_* of the PP‐TENG device. The RIGOL DHO1102 digital oscilloscope was used to collect the *V_oc_* generated by PP‐TENG. The mechanical motor provided continuous mechanical power for PP‐TENG operation.

## 2 Results and Discussion

### 2.1 The Working Mechanism of PP‐TENG Device

**Figure 2:**
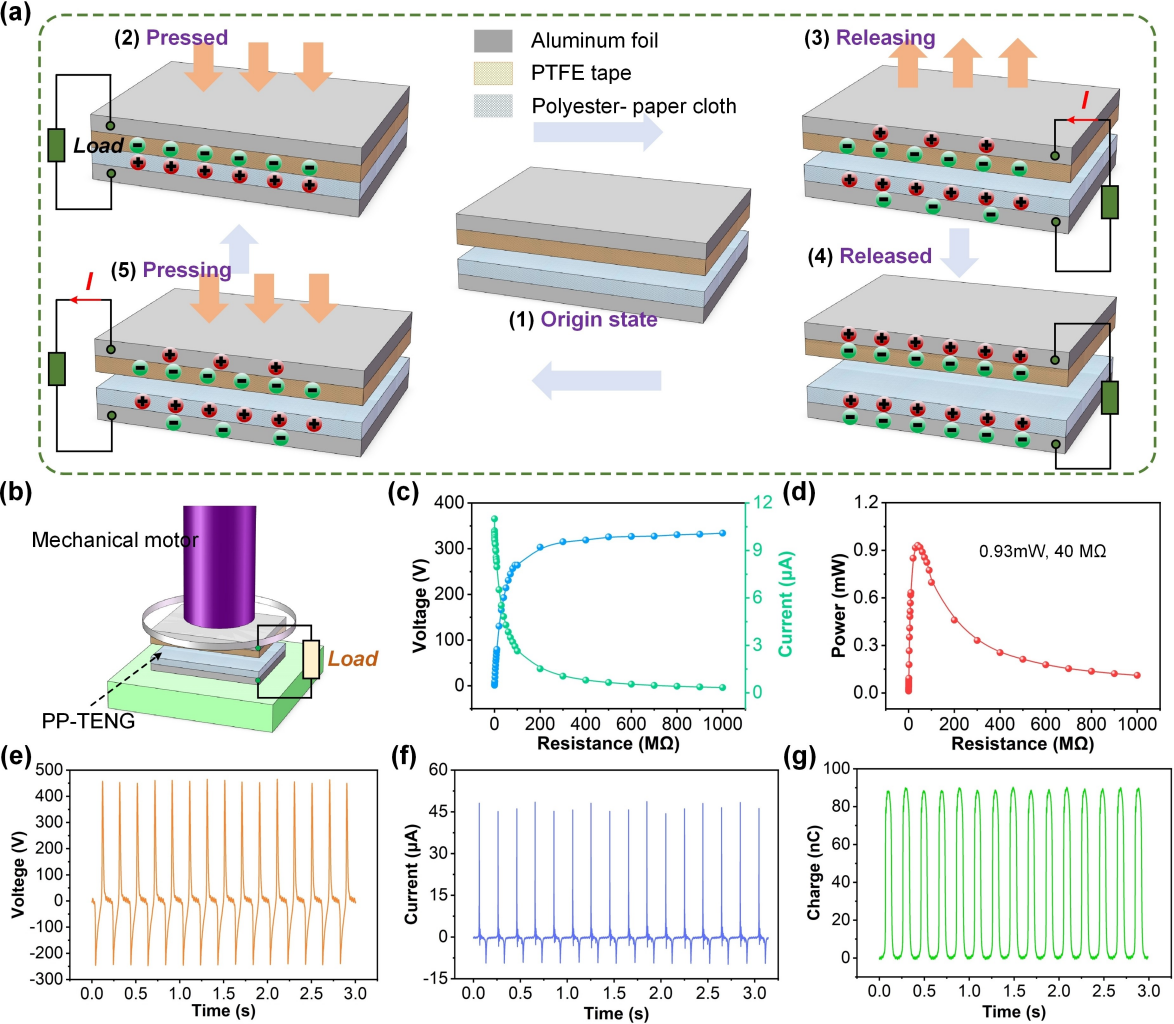
(a1–a4) The operating principle of PP‐TENG device. (b) Schematic diagram of PP‐TENG electrical testing. (c, d) The electric output of PP‐TENG with different loads. The (e) *V_oc_*, (f) *I_sc_*, and (g) *Q_sc_* of PP‐TENG under 5 Hz working frequency.

The working mode of PP‐TENG device follows a contact separation model, with no charge on the PTFE tape surface and polyester‐paper cloth surface at the beginning (Figure 2(a1)). When the external force PP‐TENG drives the PTFE tape to come into contact with the polyester paper cloth, electrons will migrate from the surface of the polyester paper cloth to the surface of the PTFE tape due to the strong electron affinity of the PTFE tape (Figure 2(a2)). After the external force is withdrawn, PP‐TENG will drive the separation of PTFE tape and polyester‐paper cloth surface due to the elastic effect of the flexible substrate, thereby generating induced current in the external circuit to balance the potential difference between PTFE tape and polyester‐paper cloth surface (Figure 2(a3)). Until the separation gap between PTFE tape and polyester paper cloth reaches its maximum, the potential system of PP‐TENG is in equilibrium and will not generate induced current (Figure 2(a4)). When PTFE tape and polyester‐paper cloth approach again under external force, the current will flow from the back electrode of PTFE tape to the back electrode of polyester‐paper cloth, thereby generating reverse current (Figure 2(a5)).

### 2.2 The Electrical Performance of PP‐TENG Device

Figure 2(b) shows the electrical performance testing system of PP‐TENG. The output current curves under different loads show that as the load increases, the output current decreases while the output voltage shows an opposite trend (Figure 2(c)). The intersection of the output current and output voltage is the maximum output power of PP‐TENG. Figure 2(d) illustrates the variation curve of PP‐TENG output power under different loads, and it can be observed that there is an optimal resistance (40 MΩ) that enables PP‐TENG to reach its maximum power of 0.93 mW. Considering that the size of PP‐TENG is 6 cm^2^, the output power density of PP‐TENG is 0.155 mW/cm^2^. Moreover, at a working frequency of 6 Hz, the *V_oc_* of PP‐TENG can reach 466.64 V, according to results in Figure 2(e). The *I_sc_* of PP‐TENG can arrive at 48.73 μA (Figure 2(f)), which can provide sufficient electrical energy for low power electronic devices. The *Q_sc_* of PP‐TENG can get to 90 nC from the results in Figure 2(g). According to Table. 1, the output performance of PP‐TENG is better than previous works.[^43^, ^44^, ^45^, ^46^]

**Table 1:** The performance comparison of PP‐TENG with previous similar works.[^43^, ^44^, ^45^, ^46^]

TENG	Fabric TENG^[43]^	Eco‐friendly TENG^[44]^	A‐TENG^[45]^	Wearable TENG^[46]^	This work
Triboelectric pair	PTFE tubes @Silk fabric	PVDF@Silver fabric	PDMS@Bare fabric	PTFE@PNA fabric	PTFE@Polyester‐paper cloth
V_oc_	100 V	105 V	49 V	36 V	466.64 V
I_sc_	6 μA	5.39 μA	1.8 μA	0.7 μA	48.73 μA
Output power density	3 μW/cm^2^	83 μW/cm^2^	9.5 μW/cm^2^	8.8 μW/cm^2^	155 μW/cm^2^

**Figure 3:**
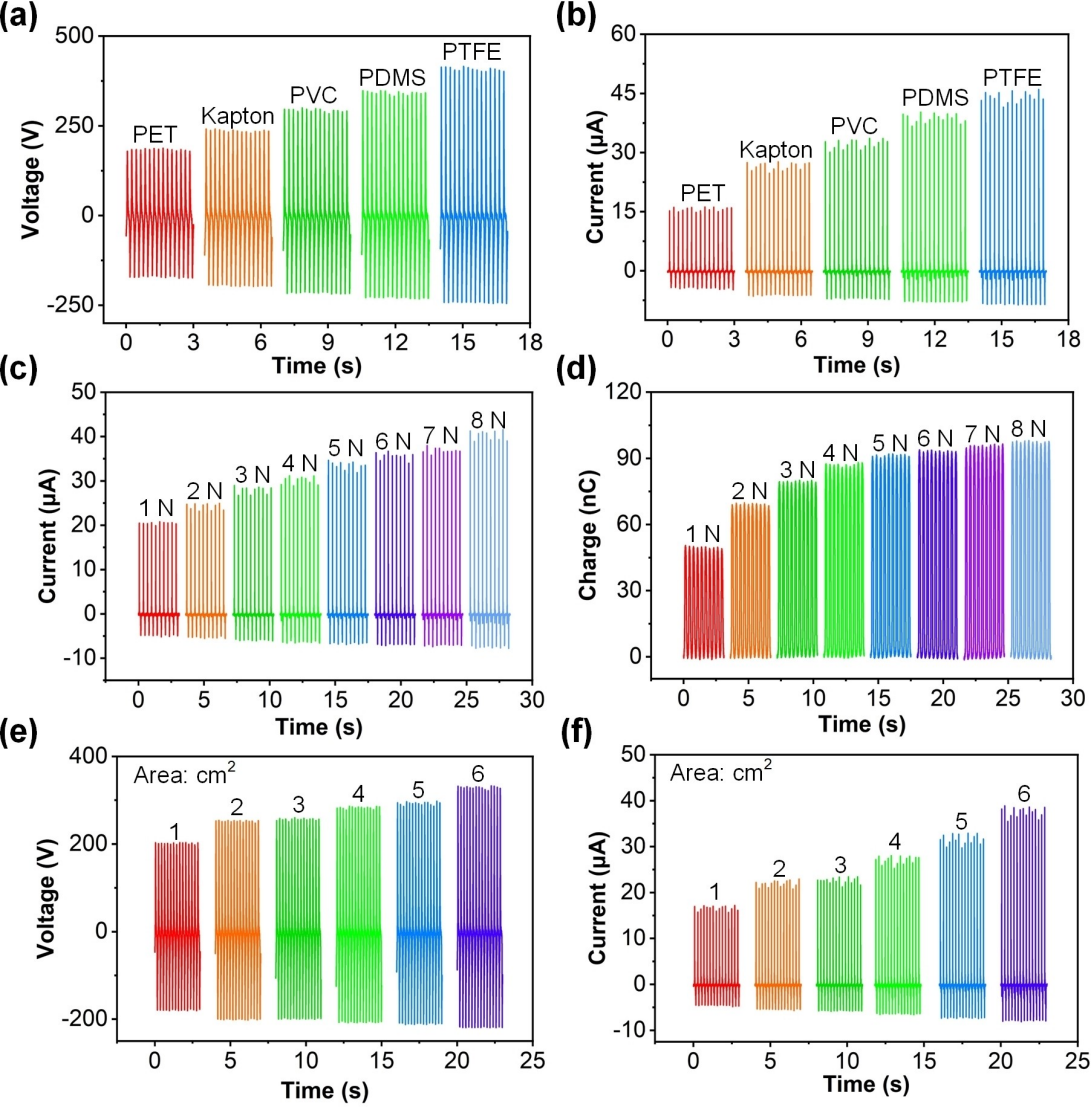
The (a) *V_oc_* and (b) *I_sc_* of TENGs with the triboelectric pair of polyester‐paper cloth@PET, polyester‐paper cloth@kapton, polyester‐paper cloth@PVC, polyester‐paper cloth@PDMS, and polyester‐paper cloth@PTFE. The (c) *I_sc_* and (d) *Q_sc_* of PP‐TENG under different pressures (from 1 N–8 N). The (e) *V_oc_* and (f) *I_sc_* of TENGs with different region area from 1 cm^2^–6 cm^2^.

The triboelectric electricity is determined by many factors such as the material's ability to gain or lose electrons, surface roughness, and affinity between materials. Based on this, we studied the electrical output performance of the combination of polyester‐paper cloth and different triboelectric materials to find the optimal triboelectric pair combination. For example, the *V_oc_* of TENGs with the triboelectric pair of polyester‐paper cloth@PET, polyester‐paper cloth@kapton, polyester‐paper cloth@PVC, polyester‐paper cloth@PDMS, and polyester‐paper cloth@PTFE can arrive at 188.11 V, 241.38 V, 300.19 V, 349.49 V, and 419.25 V, from the results in Figure 3(a). The *I_sc_* of TENGs with the triboelectric pair of polyester‐paper cloth@PET, polyester‐paper cloth@kapton, polyester‐paper cloth@PVC, polyester‐paper cloth@PDMS, and polyester‐paper cloth@PTFE can arrive at 16.34 μA, 27.71 μA, 33.72 μA, 40.29 μA, and 46.36 μA. Hence, therefore, the combination of polyester‐paper cloth and PTFE tape is due to the majority of triboelectric materials, reflecting the advantages of the combination. Due to the soft fiber structure of polyester‐paper cloth, PP‐TENG is endowed with pressure response function. The results in Figure 3(c) indicate that as the pressure increases from 1 N–8 N, the *I_sc_* of PP‐TENG also increases linearly from 21.29 μA–41.56 μA. The same trend of change also occurs in *Q_sc_* of PP‐TENG (Figure 3(d)), which is due to the fact that when polyester‐paper cloth is subjected to pressure, the fibers in the polyester‐paper cloth are compressed, resulting in an increase in the triboelectric region in PP‐TENG, which in turn leads to an increase in the electrical output of PP‐TENG. The triboelectric region is the key factor affecting the TENG output PP‐TENG, and the expansion of the triboelectric region will provide higher output for PP‐TENG. Figure 3(e) illustrates the *V_oc_* of PP‐TENG under the triboelectric region area of 1 cm^2^, 2 cm^2^, 3 cm^2^, 4 cm^2^, 5 cm^2^, and 6 cm^2^, with 204.66 V, 255.15 V, 260.21 V, 286.85 V, 298.18 V, and 333.21 V, respectively. When the triboelectric region area is 1 cm^2^, 2 cm^2^, 3 cm^2^, 4 cm^2^, 5 cm^2^, and 6 cm^2^, the *I_sc_* of PP‐TENG can reach 17.22 μA, 22.94 μA, 23.46 μA, 28.06 μA, 32.89 μA, and 38.12 μA, respectively, as illustrated in Figure 3(f). Hence, the increase of triboelectric region area is an effective means to improve PP‐TENG.

**Figure 4:**
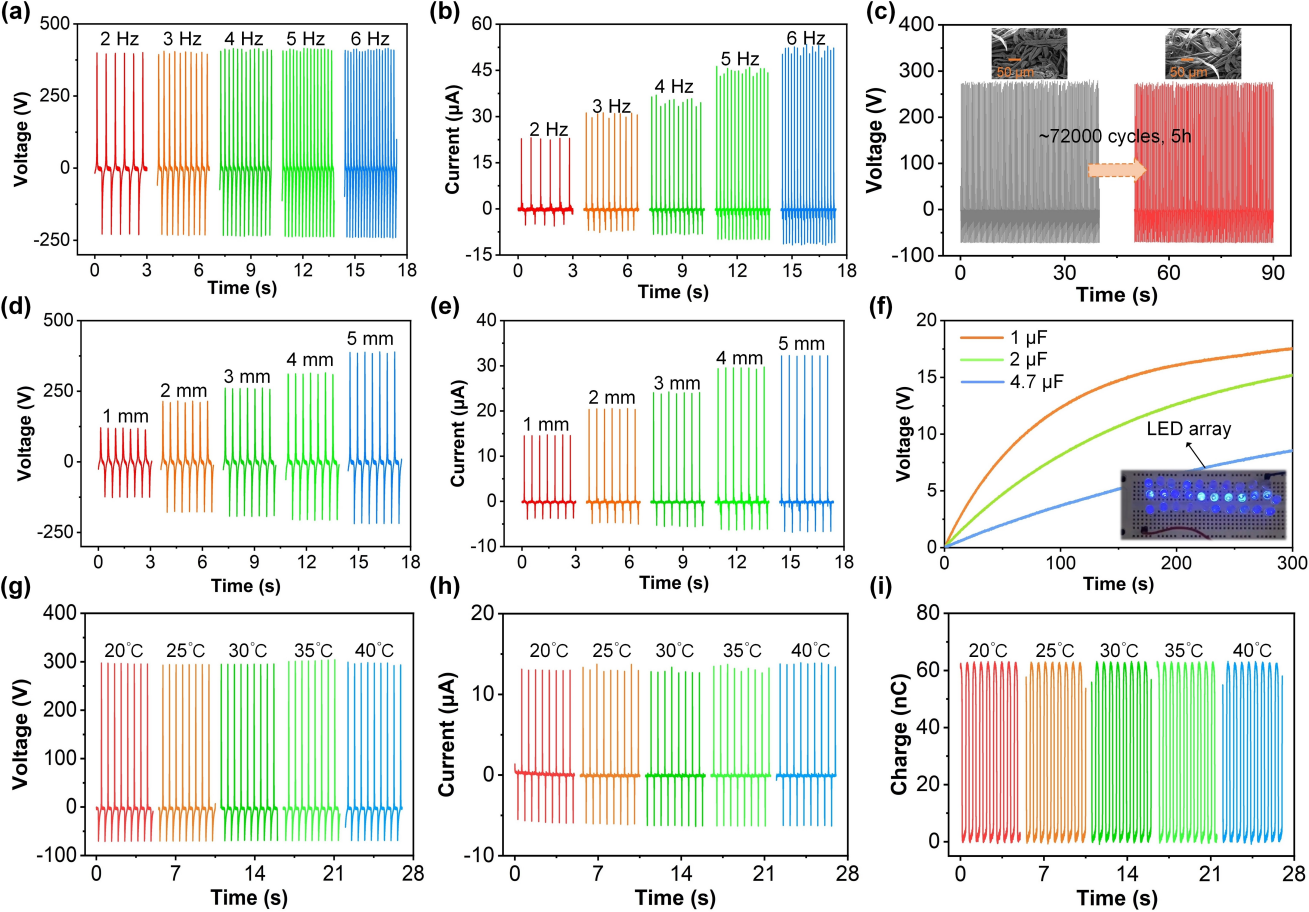
The (a) *V_oc_* and (b) *I_sc_* of PP‐TENG device with various frequencies. (c) The long‐term durability testing of PP‐TENG electrical output, inset: the SEM image of polyester‐paper cloth surface before and after 72000 working cycles. The (d) *V_oc_* and (e) *I_sc_* of PP‐TENG device with different separation distance between PTFE tape surface and polyester‐paper cloth surface. (f) The charging effect of PP‐TENG on commercial capacitors with different capacitance values, inset: 31 commercial LEDs illuminated by PP‐TENG. The (g) *V_oc_*, (h) *I_sc_*, and (i) *Q_sc_* of PP‐TENG device under different temperatures from 20 °C–40 °C.

The frequency of contact and separation between PTFE tape and polyester‐paper cloth surface will affect the PP‐TENG output. Figure 4(a) illustrates that the *V_oc_* of PP‐TENG with different frequencies from 2 Hz–6 Hz is maintained at around 410 V. However, the *I_sc_* of PP‐TENG can get to 23.16 μA, 31.26 μA, 36.99 μA, 46.31 μA, and 53.53 μA, when the working frequency is 2 Hz, 3 Hz, 4 Hz, 5 Hz, and 6 Hz. The contact and separation frequency between PTFE tape and polyester‐paper cloth surfaces will affect the transfer rate of charges in the back electrodes of the two triboelectric materials. Therefore, the working frequency factor will mainly affect the *I_sc_* of PP‐TENG. Besides, the long‐term stability of the PP‐TENG electrical output were also developed. As illustrated in Figure 4(c), no significant decreases in *V_oc_* of PP‐TENG were observed even after 32000 cycles, demonstrating that PP‐TENG is fit for mechanical energy needing a long lifetime with excellent cycling strength features.

**Figure 5:**
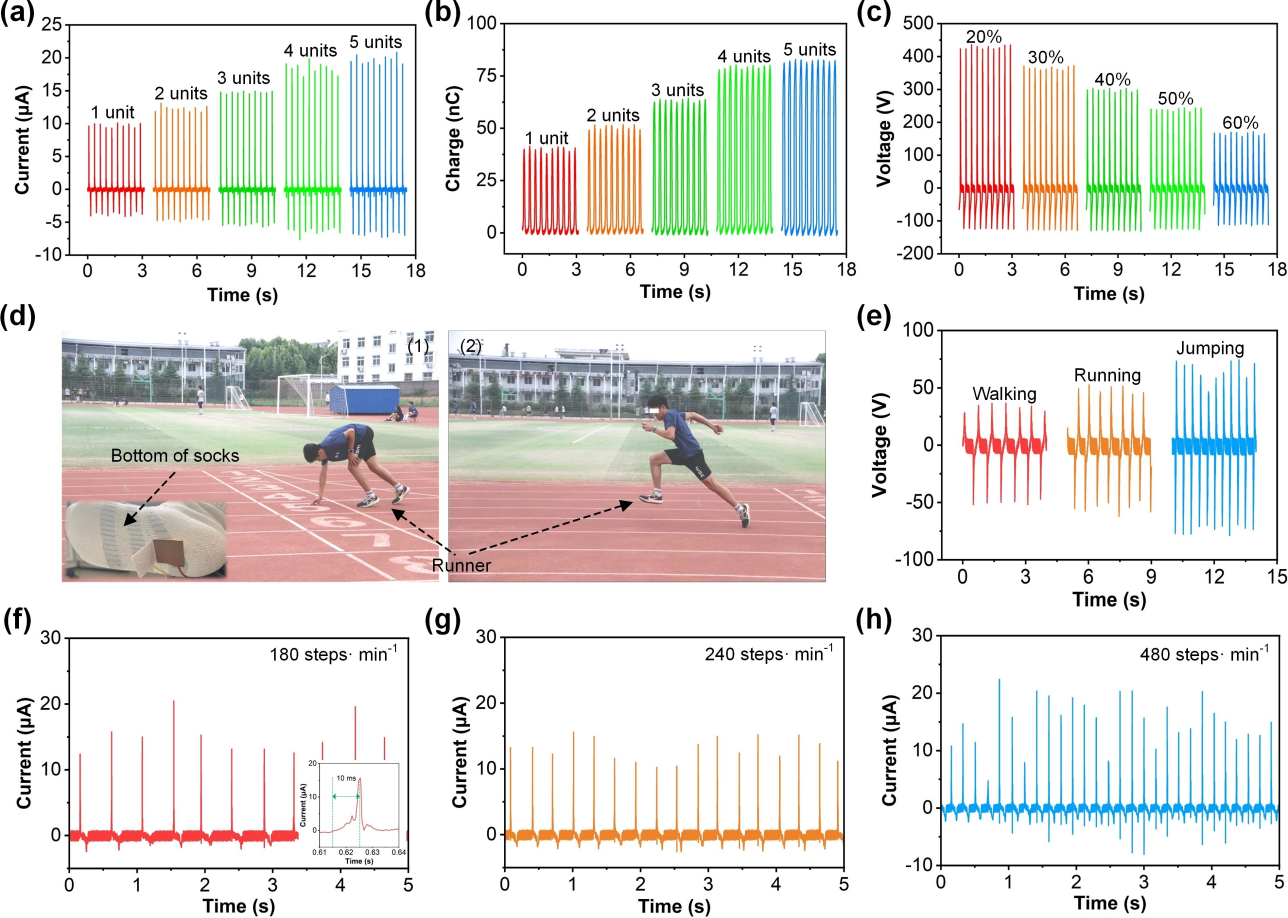
The (a) *I_sc_* and (b) *Q_sc_* of multi‐layer folding structure PP‐TENG with different number of work units. (c) The *V_oc_* of PP‐TENG under different humidity environment. (d1, d2) The pictures of athletes running, inset: the picture of installation position about PP‐TENG sensor. (e) The sensing signal of PP‐TENG under different gaits of athletes. (f‐h) PP‐TENG sensing signals of athletes at different running speeds, inset: response time of PP‐TENG sensor.

After 5 hours of continuous operation, the micro surface of the polyester‐paper cloth in PP‐TENG still showed reliability and no damage occurred, as illustrated in inset of Figure 4(c). Moreover, the separation distance between PTFE tape and polyester‐paper cloth surface will influence the PP‐TENG output, because the separation distance influences the potential difference between two triboelectric material surfaces. The results that the *V_oc_* of PP‐TENG can reach 120.99 V when the maximum separation gap is 1 mm, as illustrated in Figure 4(d). When the maximum separation gap increases to 5 mm, the *V_oc_* of PP‐TENG will also increase to 389.61 V. The same influence also occurs in the *I_sc_* of PP‐TENG, as the separation gap on the surface of PTFE tape and polyester paper cloth increases from 1 mm–5 mm, the *I_sc_* of PP‐TENG will also increase to 32.29 μA, as illustrated in Figure 4(e). Due to the fact that the electrical output generated by PP‐TENG is AC, PP‐TENG cannot directly provide electrical energy to electronic devices. In this study, PP‐TENG was rectified and charged to commercial capacitors of different capacities, including 1 μF, 2 μF, and 4.7 μF. Figure 5(f) illustrates the charging behavior of capacitors with different capacitors (1 μF, 2 μF, and 4.7 μF) when connected to the TENGs. The voltage across each capacitor increases over time, showcasing the TENG's capability to store harvested energy. The capacitor with the smallest capacitance (1 μF) charges the fastest, reaching its maximum voltage in the shortest time. This is due to the lower energy requirement to achieve a given voltage in a smaller capacitance. Although the 4.7 μF capacitor takes the longest time to charge, it can store the most energy. The energy stored in a capacitor is given by W=CV^2^/2. Thus, the larger the capacitance, the greater the energy storage potential, even if the charging rate is slower. The voltage saturation levels differ among the capacitors. The 1 μF capacitor quickly reaches a higher voltage compared to the 2 μF capacitor and 4.7 μF capacitor. This indicates that for applications requiring higher voltage but lower energy storage, smaller capacitors may be preferred. Conversely, for applications demanding significant energy storage, larger capacitors, despite their slower charging rate, would be more suitable. The PP‐TENG demonstrates effective charge storage capabilities, with the choice of capacitor size influencing the balance between charging speed and energy storage capacity. Also, 31 series connected light‐emitting diodes (LEDs) are illuminated by PP‐TENG device. This analysis underscores the versatility of PP‐TENGs in various applications, depending on the specific energy and power requirements. Figure 4(g) shows the voltage output, Figure 4(h) depicts the current output, and Figure 4(i) presents the charge output, all measured over time at temperatures ranging from 20 °C–40 °C. The results demonstrate that the PP‐TENG maintains a consistent and stable electrical output across a range of temperatures, underscoring its robust performance. This stability is essential for wearable applications, ensuring reliable energy harvesting from body movements under fluctuating thermal conditions. The device's ability to produce consistent output even at elevated temperatures further highlights its potential for integration into wearable devices, offering a sustainable power source that can operate effectively in diverse environmental conditions.

Furthermore, we investigated the electrical output of the multi‐layer folding structure PP‐TENG with multiple PP‐TENG units. The working size of each PP‐TENG unit is 2 cm×3 cm. The results in Figure 5(a) indicates the multi‐layer folding structure PP‐TENG based on 1 unit, 2 units, 3 units, 4 units, and 5 units, with the *I_sc_* of 10.48 μA, 13.48 μA, 15.21 μA, 19.85 μA, and 20.96 μA. The *Q_sc_* of the multi‐layer folding structure PP‐TENG can reach 42 nC, 54 nC, 67 nC, 81 nC, and 85 nC, corresponding to 1 unit, 2 units, 3 units, 4 units, and 5 units, as presented in Figure 5(b). We further investigated the effect of ambient relative humidity on the PP‐TENG output. Using the homemade humidity chamber, we measured the PP‐TENG electrical output under different humidity conditions. As shown in Figure 5(c), when the ambient relative humidity increased from 20 %–60 %, the V_oc_ of the PP‐TENG decreased from 437.93 V–172.57 V. This decrease is attributed to the fluffy structure of the polyester‐paper cloth, which has an affinity for water vapor. The flexible PP‐TENG device was constructed using PTFE tape, a flexible substrate, and polyester‐paper cloth as the main components. Running is one of the most popular aerobic exercises worldwide, however, incorrect running posture and gait can lead to sports injuries. Traditional sports monitoring devices typically rely on external power sources, which can lead to issues such as short battery life and inconvenience in wearing. TENG sensors have the function of self powering by converting mechanical energy into electrical energy, greatly improving the portability and service life of the equipment. Its high sensitivity enables the TENG sensor to capture small mechanical changes during running, providing accurate gait analysis, monitoring foot pressure distribution, and collecting exercise energy. Applying TENG sensors to running not only enables real‐time monitoring of runner's exercise data, but also provides scientific exercise guidance and health management recommendations. Therefore, we applied PP‐TENG to running and used the sensing signals generated by PP‐TENG as analysis data for athletes’ running training, providing a reference basis for smart running. Figure 5(d1, d2) depict the experimental setup where the TENG is attached to the bottom of a runner's socks, enabling real‐time energy harvesting from various movements. The inset in Figure 5(d1) shows the picture of installation position about PP‐TENG sensor (size: 3 cm×3 cm). Figure 5(e) demonstrates the voltage output generated during different activities: walking, running, and jumping. Walking produces lower voltage peaks compared to running and jumping, indicating a direct correlation between activity intensity and voltage generation. The increased mechanical force and frequency in running and jumping result in higher voltage outputs, highlighting the TENG's sensitivity to different motion patterns. Figure 5(f–h) further analyze the current output at different step frequencies: 180, 240, and 480 steps per minute, respectively. The inset in Figure 5(f) illustrates the response time of PP‐TENG sensor can reach 10 ms. The current output increases with step frequency, emphasizing the TENG's capability to convert kinetic energy from rapid movements into electrical energy efficiently. This trend underscores the potential of TENGs in wearable electronics, where various human activities can be harnessed to generate sustainable power. The output current frequency of PP‐TENG can reflect the athlete's step movement frequency. Specifically, the number of current signals generated by PP‐TENG is used to feedback the athlete's steps, and the number of pulses within a certain period of time is calculated to reflect the athlete's steps, which is independent of the peak output current of PP‐TENG. This periodic current pulse indicates that PP‐TENG can accurately capture changes in the pace, providing a stable output signal for real‐time monitoring and analysis of the runner's pace rate. This is of great significance for optimizing running training and monitoring the exercise status of runners.

## 3 Conclusions

In conclusion, the development and integration of the PP‐TENG mark a significant advancement in the field of wearable electronics and intelligent paper electronics. The PP‐TENG showcases superior performance metrics with an *V_oc_* of 466.64 V, a *I_sc_* of 48.73 μA, and a *Q_sc_* of 90 nC. These impressive values highlight the potential of PP‐TENG in efficient energy harvesting, especially for applications requiring high sensitivity to mechanical pressure. Besides, the maximum output power of the PP‐TENG reaches 930.26 μW when paired with a matched load of 40 MΩ. The innovative use of polyester‐paper cloth, with its inherent durability and tear resistance, addresses the limitations of traditional paper‐based TENGs, ensuring suitability for long‐term wearable applications. The integration of PP‐TENG into the soles of athletic socks demonstrates its practical utility, providing real‐time monitoring of runners’ gait and step count. The PP‐TENG's ability to convert mechanical energy from human motion into electrical energy presents a promising solution for self‐powered wearable sensors, contributing to the growing field of smart paper electronics.

## Conflict of Interests

The authors declare that they have no conflict of interest.

## Data Availability

The data that support the findings of this study are available from the corresponding author upon reasonable request.
